# Vestibular Profile of Patients with Hearing Loss Caused by Pathogenic Variants of the *STRC* Gene

**DOI:** 10.1097/ONO.0000000000000084

**Published:** 2026-01-27

**Authors:** Zuzana Balatková, Veronika Svobodová, Vladimír Koucký, Zuzana Libáková, Anna Kameníková, Dana Šafka Brožková, Martin Komarc, Markéta Bonaventurová, Vít Kruntorád, Zdeněk Čada

**Affiliations:** 1 Department of Otorhinolaryngology, 2nd Faculty of Medicine, Charles University, Motol University Hospital, Prague, Czech Republic; 2 Department of Otorhinolaryngology and Head and Neck Surgery, 1st Faculty of Medicine, Charles University, Motol University Hospital, Prague, Czech Republic; 3 DNA laboratory, Department of Paediatric Neurology, 2nd Faculty of Medicine, Charles University, Motol University Hospital, Prague, Czech Republic; 4 Department of Anthropomotorics and Methodology, Faculty of Physical Education and Sport, Charles University, Prague, Czech Republic; 5 Department of Paediatric Otorhinolaryngology, Faculty of Medicine, Masaryk University and University Hospital Brno, Brno, the Czech Republic.

**Keywords:** Audiological phenotype, Autosomal recessive nonsyndromic hearing loss, DFNB16, *STRC*, Stereocilin, Vestibular function

## Abstract

**Introduction:**

The second most frequent cause of autosomal recessive nonsyndromic sensorineural hearing loss (AR-NSHL) worldwide is a biallelic pathogenic alteration of the *STRC* (stereocilin) gene, also named DFNB16. The type and severity of hearing impairment in DFNB16 patients were studied thoroughly, while information on a detailed examination of their vestibular function is still lacking. Our aim was to characterize the vestibular status of patients with biallelic pathogenic variants in *STRC* by performing a complete up-to-date test battery.

**Methods:**

Eight AR-NSHL patients, aged 6–37 (mean age ± standard error of the mean [SEM] 16.13 ± 8.67), underwent standard audiological testing and otoneurologic investigation, including videonystagmography (VNG) with caloric stimulation, video head-impulse test (vHIT), and cervical vestibular evoked myogenic potentials (c-VEMPs). Subjects were divided into three groups (group 1, 2, and 3) according to the type of diagnosed *STRC* gene variant.

**Results:**

The grade of the hearing loss was calculated as pure tone average (PTA) (mean PTA ± SEM 41.88 dB ± 5.49). The vHIT displayed nearly normal bilateral gain and mostly the absence of saccades in all examined groups. Cervical VEMPs in response to air-conducted and bone-conducted stimuli showed prolonged latencies of waves P1 and N1 bilaterally in group 1, although latencies in groups 2 and 3 were within normal range. The results of VNG indicated normal vestibular and central oculomotor function.

**Conclusions:**

Biallelic pathogenic variants in the *STRC* gene influence the inner ear’s cochlear and vestibular function. Certain vestibular abnormalities in DFNB16 patients were detected by detailed evaluation, despite none of the DFNB16 subjects in our study reported subjective symptoms.

Vertigo or dizziness is a common symptom. The etiology comprises vestibular and nonvestibular causes. Vestibular syndromes may be of central or peripheral origin. Hence, peripheral vestibular disorders are a diverse group of disorders. They are most frequently represented by benign paroxysmal positional vertigo, Meniere’s disease, and unilateral vestibulopathy (1).

The role of inheritance in vestibular disorders has growing evidence (2). Partial genetic origin was already described in Meniere’s disease, vestibular migraine, vestibular schwannoma, enlarged vestibular aqueduct syndromes, bilateral vestibulopathy, CANVAS (cerebellar ataxia, neuropathy, and vestibular areflexia), and several types of hereditary sensorineural hearing loss with vestibular dysfunction (3). Genetic causes were identified in patients with deafness syndromes, vertigo attacks, and neurological diseases (4). Pathogenic variants in the *KCNA1* and *CACNA1A* genes (episodic ataxia type 1 and type 2) suggest that voltage-gated channels and solute carriers in the plasma membrane of neurons play a key role in recurrent vertigo (5). A genome-wide association study uncovered 6 sequence variations associated with vertigo risk (6). The central nervous system also plays an important role in the pathogenesis of vertigo (7). However, detailed characteristics of the genetics of vertigo remain unclear.

Stereocilin is a protein associated with the hair bundle of the sensory hair cells in the inner ear. It is encoded by the *STRC* gene in humans. Stereocilin is expressed in the stereocilia of cochlear outer hair cells, where it is essential for mechanoelectrical transduction, and in the kinocilia of the vestibular sensory organs (utricle, saccule, and semicircular canals). These structures enable the perception of motion. This dual expression suggests that pathogenic variants may affect both auditory and vestibular function. The *STRC* gene is a part of a tandem duplication on chromosome 15 (15q15); the second copy is a pseudogene (8). The impact of *STRC* causal variants on the cochlea has already been thoroughly studied (9,10). This condition leads to DFNB16 autosomal recessive nonsyndromic sensorineural hearing loss (AR-NSHL). The role of stereocilin in the vestibular apparatus and its clinical consequences for potential dizziness remain to be elucidated. According to the brief literature, disequilibrium in 48 families with DFNB16 type of deafness was reported, but proper diagnosis of vestibulopathy was missing (11). Mostly, paroxysmal and positional dizziness or vertigo were described in patients with known *STRC* pathogenic variants (12–14). Nevertheless, the precise assessment of their vestibular profile using current standard otoneurological methods is still lacking.

## MATERIAL AND METHODS

The study was approved by the Ethics Committee of the hospital. All the patients or their parents signed an informed consent to attend the study. Eight patients with DFNB16 and early hearing impairment participated in the study, 3 male and 5 female, aged 6–37 (mean age ± standard error of the mean [SEM] 16.13 ± 8.67). Patients underwent structured family and personal history examinations and claimed no history of vestibular symptoms nor disequilibrium. Subjects were divided into 3 groups according to *STRC* pathogenic variant type: group 1—homozygous STRC deletions; group 2—compound heterozygous nonsense variants; group 3—heterozygous deletion combined with a hemizygous nonsense variant (Table 1). The first group consisted of 4 patients with homozygous deletions of the *STRC* gene. The second group was comprised of 2 patients with compound heterozygous pathogenic point variants, and the third group was composed of 2 patients with a combination of heterozygous deletion and hemizygous pathogenic point variant.

**TABLE 1.** The spectrum of detected STRC mutations in the DFNB16 patients

No.	Age/sex	Allele 1	Allele 2	Variant interpretation	vHIT (gain/saccades)	VNG	cVEMP (P1/N1 latency, AAR)
1	37/F	STRC deletion	STRC deletion	Homozygous deletion	Normal, no saccades	Normal	Prolonged P1/N1, AAR ≤ 16%
2	10/F	STRC deletion	STRC deletion	Homozygous deletion	Normal	Normal	Prolonged P1/N1, AAR ≤ 16%
3	13/F	STRC deletion	STRC deletion	Homozygous deletion	Normal	Normal	Prolonged P1/N1, AAR ≤ 16%
4	17/M	STRC deletion	STRC deletion	Homozygous deletion	Normal	Normal	Prolonged P1/N1, AAR ≤ 16%
5	15/F	c.4402C>T (p.Arg1468*)	c.3217C>T (p.Arg1073*)	Compound heterozygous nonsense variants	Normal	Normal	Normal latencies
6	18/M	c.4402C>T (p.Arg1468*)	c.3217C>T (p.Arg1073*)	Compound heterozygous nonsense variants	Normal	Normal	Normal latencies
7	6/F	STRC deletion	c.3217C>T (p.Arg1073*)	Heterozygous deletion + hemizygous nonsense variant	Slightly low posterior gain, saccades present	Normal	Abnormal AAR up to 46%
8	13/M	STRC deletion	c.3217C>T (p.Arg1073*)	Heterozygous deletion + hemizygous nonsense variant	Slightly low posterior gain, saccades present	Normal	Abnormal AAR up to 46%

AAR indicates amplitude asymmetry ratio; c-VEMPs, cervical vestibular evoked myogenic potentials; vHIT, video head-impulse test; VNG, videonystagmography.

An otoscopic examination, confirming an intact tympanic membrane, was performed on all the subjects. Audiologic tests were carried out, including pure tone audiometry (audiometer Madsen Astera, Otometrics, DK, in frequencies 250, 500, 1000, 2000, 4000, and 6000 Hz), tympanometry, and stapedial reflexes (tympanometer AZ 26, AT 35, Interacoustics, DK).

Subjects underwent a complete vestibular test battery, including videonystagmography (VNG) with caloric air stimulation, video head-impulse test (vHIT), and cervical vestibular evoked myogenic potentials (c-VEMPs) in response to air-conducted (AC) and bone-conducted (BC) stimuli. Rotational testing was not used. VNG was performed with a video VNG analyzer with comprehensive software (GN Otometric, Denmark). The measurement comprised spontaneous nystagmus with and without fixation, gaze direction nystagmus, smooth pursuit test, examination of saccades, recording of optokinetic nystagmus, and caloric tests. Presence of nystagmus and alteration of latency, gain, and accuracy were monitored. To assess the reactivity of all 6 semicircular canals, the subjects were examined by vHIT using high-frequency passive head acceleration (EyeSeeCam vHIT, Interacoustics, Denmark). Vestibulo-ocular reflex gain was calculated as the regression slope between eye and head velocity. The normative range lies between 0.7 and 1.2 (15). AC and BC c-VEMPs were recorded (Eclipse, Interacoustics, Denmark). Electrodes were placed on the border of the cranial and middle third of the sternocleidomastoid muscle each time. The measurements were performed using insert phones for air conduction and a B81 vibrator for bone conduction to obtain latency and amplitude of P1 and N1 waves of the left and right otolith organ, and to evaluate their potential asymmetry. Normal latency values of the AC c-VEMPs response were considered to be 13 ms for P1 and 23 ms for N1. The stimuli were 95 dB HL tone burst (500 Hz) and 500 Hz vibration at 60 dB HL. The amplitude asymmetry ratio (AAR) was assessed; AAR> 0.35 was considered to indicate asymmetry between the 2 sides based on the normative values from the literature (16), which matched the results of our laboratory as well.

### Genetic Testing

In patients with early hearing loss, genetic testing was performed as described previously (10) in the following order: first, we used Sanger sequencing to exclude biallelic pathogenic variants in GJB2. Next, to exclude *STRC* gene deletions, we performed quantitative comparative fluorescent polymerase chain reaction (QCF-PCR). Homozygous and heterozygous deletions detected by QCF-PCR were confirmed by an independent method: multiplex-ligation probe amplification (MRC-Holland, Netherlands). Patients with heterozygous STRC deletion and patients without deletion were subjected to a targeted gene panel testing of genes associated with hearing loss. Sure-Select target enrichment kit (Agilent Technologies, US) was used for library preparation and sequenced on the Illumina Hi-Seq platform (Illumina, Inc., US). Two independent bioinformatic pipelines were utilized for Fastq data analysis (NextGene [Softgenetics, US] and SureCall [Agilent Technologies, US]). To verify the presence of the detected point variants in the *STRC* gene, a specific product without contamination of the pseudogene pSTRC was generated by long-range PCR (17).

### Statistical Analysis

Descriptive statistics were employed to analyze the data, focusing on summarizing key trends and distributions. Measures of central tendency and variability, including the mean and SEM, were calculated to represent the average values and spread of the variables of interest. Due to the limited sample size, inferential statistics were not applied, as the analysis was primarily exploratory. The analysis was conducted using SPSS version 25 (SPSS Inc., Chicago, IL, USA), and results are presented in Tables and Figures to provide a clear overview of the key findings (18).

## RESULTS

**FIG.1.**
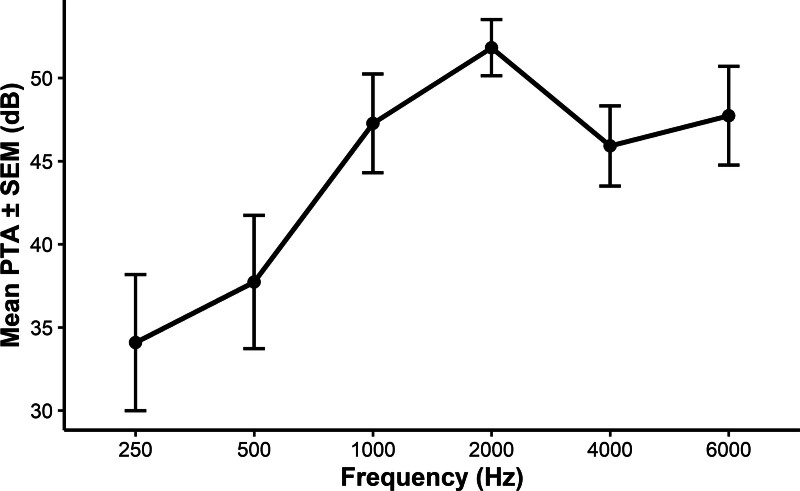
The audiological mean of PTA ± SEM among *STRC* gene mutation subjects. PTA indicates pure tone average; SEM, standard error of the mean.

**FIG. 2.**
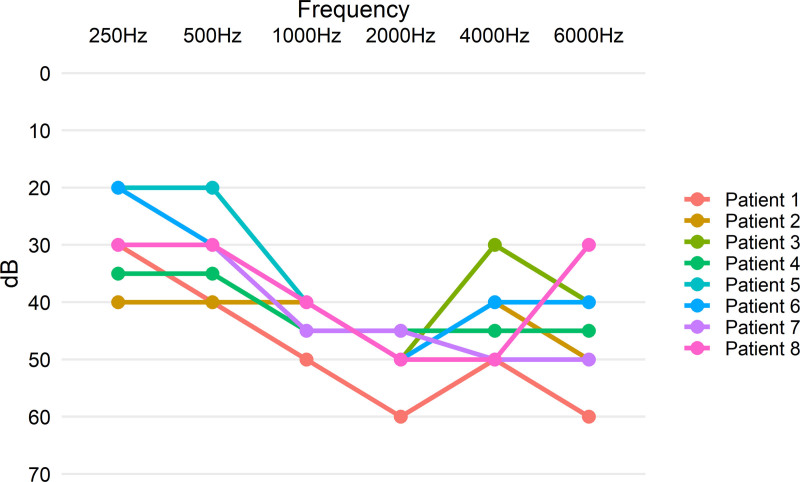
Individual audiograms of the subjects. PTA indicates pure tone average.

Pure tone average (PTA) was calculated to grade the level of hearing loss from frequencies 500, 1000, 2000, and 4000 Hz (mean PTA ± SEM 41.88 dB ± 5.49). The results corresponded with common DFNB16-associated hearing loss, which can be characterized as a moderate sensorineural hearing loss with the cup-shaped deflection of the auditory curves at mid frequencies (Fig. 1) (10). Individual audiograms can be found in Figure 2.

The vHIT test showed a normal bilateral gain and no presence of saccades in groups 1 and 2 (>0.7 in all 6 semicircular canals) and nearly normal results in the case of group 3 (with the exception of low normal gain >0.62 in only the left posterior semicircular canal and the presence of overt and covert saccades) (15).

**FIG. 3.**
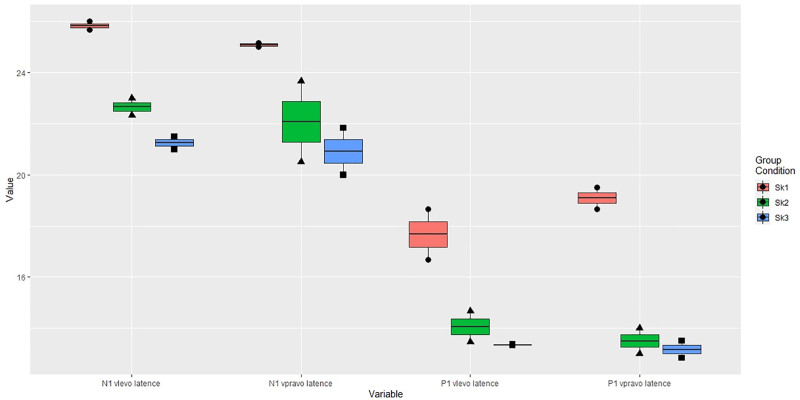
Air-conducted c-VEMPs showing prolonged latencies of waves P1 and N1 in group 1. C-VEMPs indicates cervical vestibular evoked myogenic potentials.

**FIG. 4.**
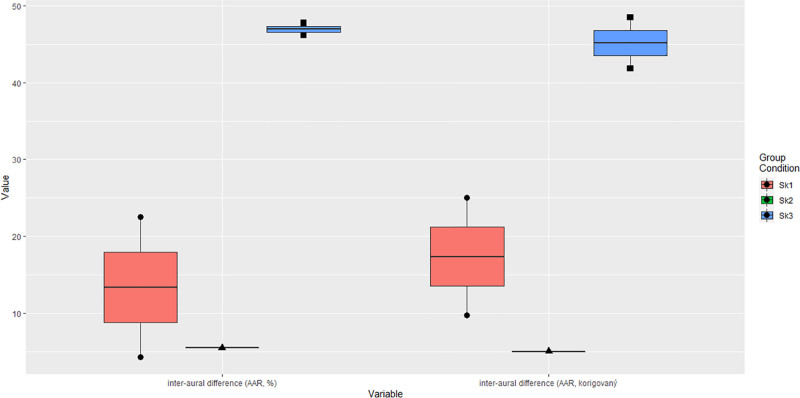
Higher asymmetry ratio after correction in group 3. AAR indicates amplitude asymmetry ratio.

There was a response present for cervical AC and BC VEMPs in all tested groups. The c-VEMPs with AC acoustic stimulation showed prolonged latencies of waves P1 (>13 ms) and N1 (>23 ms) bilaterally in group 1 (P1 mean latency ± SEM 18.38 ms ± 1.04, N1 mean latency ± SEM 25.46 ms ± 0.40), although latencies in groups 2 and 3 were within normal range (Fig. 3). Amplitudes were similar on both sides after correction, and the asymmetry ratio of interaural difference reached the maximum of 16.5 % in all patients. The values obtained by performing BC c-VEMPs were analogous and showed prolonged latencies of waves P1 and N1 in group 1 (P1 mean latency ± SEM 16.70 ms ± 2.57, N1 mean latency ± SEM 24.27 ms ± 1.56). The singular difference was displayed in the asymmetry ratio in group 3, where the values of BC c-VEMPs were pathologically higher, up to 46.1% (Fig. 4).

The results of VNG indicated normal vestibular and central oculomotor function in general (19). No spontaneous nystagmus (with or without fixation), nor gaze direction nystagmus was detected. Normal gain values were reached in the smooth pursuit test, and examination of saccades demonstrated physiological accuracy, peak velocity, and latency of the saccades. Recordings of optokinetic nystagmus were symmetrical on both the right and the left side, with normal values of peak slow-phase velocity. Air calorics showed normal total responses, directional preponderance, and gain asymmetry.

## DISCUSSION

The *STRC* gene pathogenic variants are primarily associated with AR-NSHL, typically moderate, with the cup-shaped deflection of the auditory curves at mid frequencies. This is the second most common cause of AR-NSHL worldwide (20). The impact of stereocilin dysfunction on the vestibular apparatus and state of equilibrium has not been completely identified yet. Sporadic evidence from single patients indicates an incomplete vestibular lesion in carriers of DFNB16 (14,21).

In this study, we aimed to precisely characterize vestibular function and its clinical consequences within a group of patients with 3 types of stereocilin variants. These groupings reflect different variant types and were intended to explore whether mutation type may influence vestibular phenotype. However, given the limited cohort size, these comparisons should be interpreted with caution. Although none of the 8 patients claimed any vestibular symptoms, certain abnormal values of examined parameters were detected. Control patients were not included due to the rarity of STRC-positive cases. Instead, we compared our findings to published data from other genetic hearing loss groups. For example, vestibular dysfunction in GJB2 patients is variable, while other genes (eg, OTOF, TMC1) have not been linked to peripheral vestibular dysfunction. This contextualizes our findings within the broader field of genetic hearing loss.

Stereocilin is located in the hair cells of the cochlea and in the vestibular receptor organs of semicircular canals (ampullary crests) and utricle and saccule (maculae). In animal models, stereocilin is predominantly expressed in the saccule and utricle, suggesting a role in vestibular sensory transduction. Its localization to the kinocilia of vestibular hair cells indicates that STRC dysfunction may impair peripheral vestibular signaling, although central compensatory mechanisms may mask clinical symptoms. Our findings of subclinical abnormalities support this hypothesis (22). Accordingly, parameters obtained by vestibular testing showed only limited abnormality (prolonged latencies of waves P1 and N1 of AC c-VEMPs in group 1), depending primarily on peripheral parts of the vestibular apparatus, and this dysfunction was revealed by detailed vestibular investigation. Results of vHIT were low normal, and vestibulo-collic and vestibulo-ocular reflexes evaluated by VEMPs showed different responses within the 3 tested groups. One of the most interesting findings was the prolongation of latencies of waves P1 and N1 in group 1, while latencies in groups 2 and 3 stayed within the normal range. This suggests a possible variable extent of impairment according to the detected variant type, differing in the case of complete gene deletion and its altered form. The division into 3 groups was exploratory, aiming to observe whether variant type could be associated with different vestibular phenotypes. This approach is limited by small cohort size and should be interpreted cautiously, without implying distinct pathomechanisms. Although none of the patients reported vestibular symptoms, prolonged P1 and N1 latencies in c-VEMPs may reflect subclinical peripheral vestibular involvement. While these findings may not have direct clinical consequences in asymptomatic individuals, they contribute to understanding the vestibular phenotype of STRC-related hearing loss and may become relevant for gene therapy or translational research. The differences in audiological findings within the 3 groups are difficult to evaluate due to the small cohort size and were not the primary outcome measures of the presented study.

None of the patients in our cohort reported balance problems, but cases with biallelic pathogenic variants in the *STRC* gene and episodic positional vertigo subsiding with age can be found in the literature. Peripheral vestibular dysfunction was proved by performing VEMPs, caloric stimulation, and subjective visual horizontal test, while function captured by vHIT stayed within normal range (12). Some of the patients in other studies had vestibular symptoms, but vestibular function was not systematically investigated. In their case, vestibulopathy cannot be excluded (11,23). The definitive vestibular status is also determined by the influence of central compensatory mechanisms, which strongly impact final vestibular function. Central compensatory mechanisms are of great importance, especially in the pediatric population (24,25).

### Study Limitations

The main limitation of our study is the small cohort size, which precludes definitive conclusions. However, due to the rarity of STRC-related hearing loss, recruiting a larger group is challenging. We consider our findings to provide valuable pilot data for future multicenter or international studies.

## CONCLUSION

The possible impact of *STRC* biallelic variants on both the cochlear and the vestibular part of the inner ear was confirmed. We revealed typical moderate sensorineural hearing loss with deflection of the auditory curves at mid frequencies in agreement with the results of other authors, and limited abnormity depending primarily on peripheral parts of the vestibular apparatus in patients with homozygous deletions of the *STRC* gene, and a higher amplitude asymmetry ratio in group 3 was observed. These ought to be a sign of altered vestibular function, specific for the type of genetic mutation. Extension of studied groups and future observation of clinical manifestations are necessary to precisely describe the impact of certain mutations on the vestibular system. The results within the 3 tested groups suggest a variable extent of impairment according to the pathogenic variant type. This is one of the first studies that systematically evaluates vestibular function in DFNB16 patients using a detailed contemporary vestibular test battery.

## FUNDING SOURCES

The study was supported by the University Hospital Motol, by the Institutional Support-Modern therapy No. 9774, and by the Project for conceptual development of research organization 00064203. Open access publishing is supported by the National Technical Library in Prague.

## CONFLICT OF INTEREST

None declared.

## DATA AVAILABILITY

The data will be made available upon reasonable request.

## ETHICAL CONSIDERATION

A local ethics committee approved the study. It was performed per the ethical standards laid down in the 1964 Declaration of Helsinki and its later amendments, with patients giving their informed consent.

## AUTHOR CONTRIBUTIONS

Conceptualization: Čada Zdeněk, Balatková Zuzana; Methodology: Balatková Zuzana; Investigation: Bonaventurová Markéta, Koucký Vladimír, Libáková Zuzana, Procházková Anna, Šafka Brožková Dana, Svobodová Veronika; Statistical analysis: Komarc Martin; Writing - original draft preparation: Balatková Zuzana, Svobodová Veronika; Writing - review and editing: Bonaventurová Markéta, Balatková Zuzana, Koucký Vladimír, Čada Zdeněk, Šafka Brožková Dana, Krunotrád Vít; Funding acquisition: Čada Zdeněk; Supervision: Balatková Zuzana, Čada Zdeněk.
